# Genomic insights into the ESBL and MCR-1-producing ST648 *Escherichiacoli* with multi-drug resistance

**DOI:** 10.1007/s11434-016-1086-y

**Published:** 2016-05-19

**Authors:** Huimin Zhang, Christopher H. Seward, Zuowei Wu, Huiyan Ye, Youjun Feng

**Affiliations:** Department of Medical Microbiology and Parasitology, Zhejiang University School of Medicine, Hangzhou, 310058 China; Carl R. Woese Institute for Genomic Biology, University of Illinois at Urbana-Champaign, Urbana, IL 61801 USA; Department of Veterinary Microbiology and Preventive Medicine, Iowa State University, Ames, IA 50010 USA

**Keywords:** MCR-1, Extended-spectrum beta-lactam (ESBL), Colistin resistance, ST648

## Abstract

Polymyxin acts as an ultimate line of refuge against the severe infections by multidrug-resistant Gram-negative pathogens. This conventional idea is challenged dramatically by the recent discovery of mobile colistin resistance gene (*mcr*-*1*) is prevalent in food animals and human beings worldwide. More importantly, the *mcr*-*1* gene was found to be co-localized with other antibiotic resistance genes, raising the possibility that super-bugs with pan-drug resistance are emerging. However, little is reported on the genomes of the *mcr*-*1*-positive bacterial host reservoirs. Here we report genome sequencing of three human isolates of the *mcr*-*1*-positive *Escherichia coli* (E15004, E15015 and E15017) and define general features through analyses of bacterial comparative genomics. Further genomic mining together with sequence typing allowed us to elucidate that the MCR-1-carrying *E. coli* E15017 belongs to the sequence type ST648 and coproduces extended-spectrum β-lactamase (ESBL). Given the fact that ST648 has been known to associate with either New Delhi metallo-β-lactamase 1 or ESBL, our results highlighted the possibility of ST648 as an epidemic clone with multidrug resistances.

**Electronic supplementary material:**

The online version of this article (doi:10.1007/s11434-016-1086-y) contains supplementary material, which is available to authorized users.

## 摘要

通常认为多粘菌素是抵御多药耐受的革兰阴性病原菌严重感染的最后一道防线。 最新研究表明在动物类食品和人体肠道菌群中存在一种多粘菌素抗性基因 (*mcr*-*1*)。这一惊人发现对上述传统观点提出了严重挑战。更为惊讶的是, 该 *mcr*-*1* 基因可与其他抗生素抗性基因共存, 极大增加了产生具有广泛耐药的超级 细菌的可能性。但是, 关于*mcr*-*1* 阳性细菌的基因组学特征鲜见报道。本研究对 3 株 *mcr*-*1* 阳性人源大肠杆菌(E15004, E15015 和E15017)开展了全基因组 测序, 并通过比较基因组学描述了其基因组学特征。基因组深入挖掘和序列分型 实验揭示了序列型为ST648 的大肠杆菌E15017 不仅编码MCR-1 多粘菌素耐药 基因, 而且携带超广谱β-内酰胺耐药基因(ESBL)。鉴于序列型ST648 的大肠杆 菌通常与新德里β-内酰胺耐药-1 (NDM-1) 或ESBL 紧密关联这一事实, 我们的 研究结果提示了具有MCR-1 和ESBL 耐药的ST648 细菌发展成流行性克隆的潜 在风险。

The identification of the mobilized colistin resistance gene *mcr*-*1* recently attracted extensive attention from the scientific community. MCR-1 confers resistance to polymyxins, a group of polypeptide antibiotics that are currently considered the last refuge of therapeutics against lethal challenges by Gram-negative pathogens with multi-drug resistance [1, 2]. Very recently, two separate groups reported the co-occurrence of MCR-1 and extended-spectrum β-lactamase (ESBL) on plasmids in Enterobacteriaceae [3–6]. However, genomic hallmarks of the bacterial host reservoir for the *mcr*-*1*-harbouring plasmids remain unclear. Here we report on their genomic compositions.

**Fig. 1:**
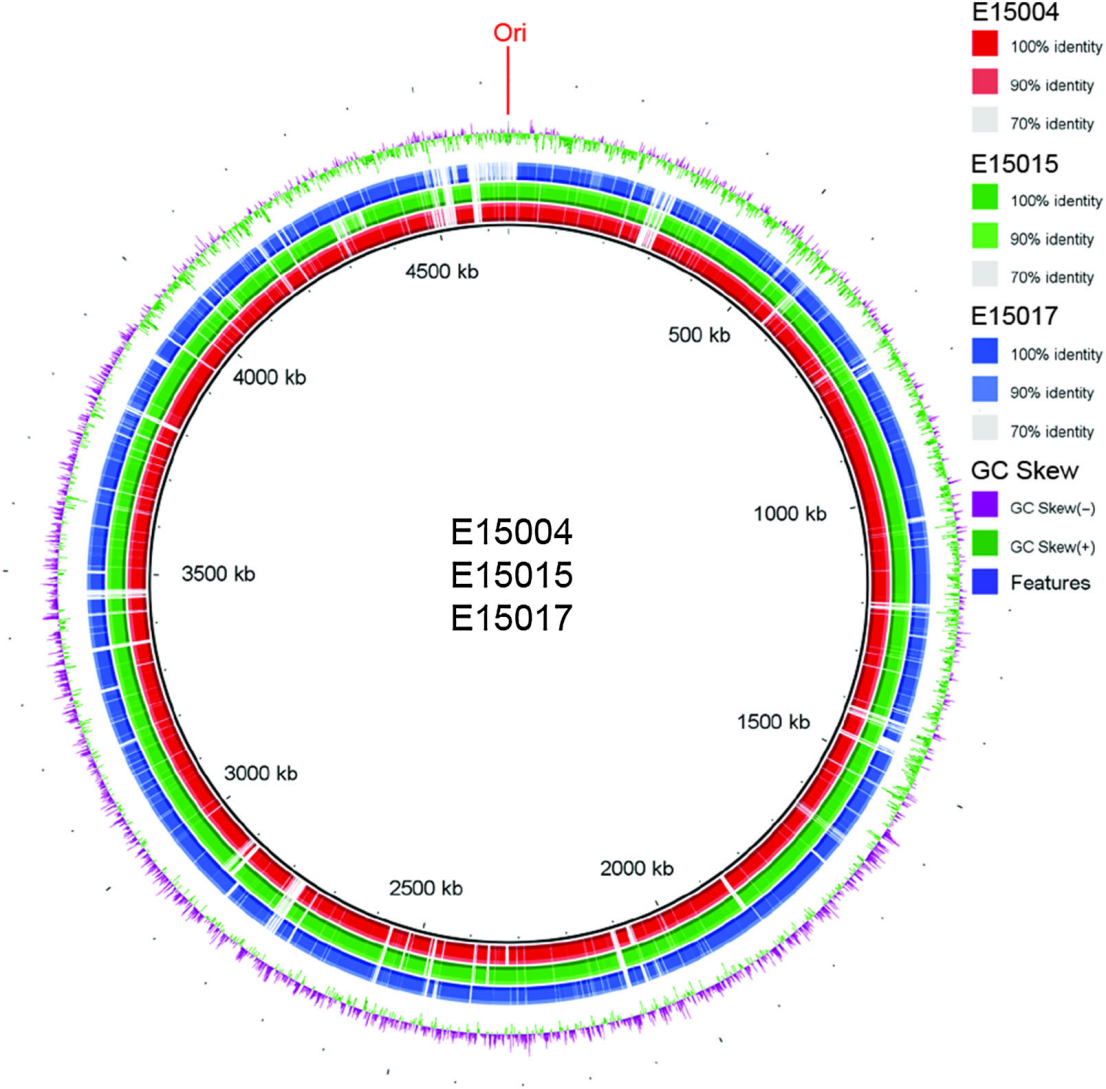
Genomics-based discovery of multidrug-resistant genes in the *mcr*-*1*-positive ST648 *E. coli* coproducing extended-spectrum β-lactamase. Circular comparison of the three sequenced genomes (E15004, E15015 and E15017) with the paradigm strain MG1655 as the reference. Individual rings range from 1 (inner ring) to 4 (outer ring). (Ring 1—red) Strain 15005 conservation plot. (Ring 2—green) Strain 15015 conservation plot. (Ring 3—blue) Strain 15015 conservation plot. (Ring 4—magenta/green) GC Skew of MG1655 reference genome [(G−C)/(G+C)] magenta > 0, green < 0

After three *mcr*-*1*-positive *E. coli* isolates (E15004, E15015 and E15017) were successfully screened from the microbiota of clinical diarrhea patients [7], we applied next-generation Illumina MiSeq sequencing to decode their genomic sequences. The pool of paired-end reads produced here were assembled with GS De Novo Assembler into a collection of contigs. Then the individual contigs were ordered into draft genomes with the prototypical strain of *E. coli* MG1655 as the reference (Fig. 1, S1). Relative to the paradigm version of *E. coli*, MG1655 (4,641,425 bp), the three *mcr*-*1*-positive clinical *E. coli* isolates exhibited variations in the size of sequenced genomes (i.e., 4,643,275 bp for strain E15004; 4,637,424 bp for strain E15015, and 4,780,540 bp for strain E15017) (Table S1). The values of their GC percentages are all approximately 50 % (Table S1), although the draft genomes identified several regions with a strong GC skew, indicative of novel insertions of genomic material.

Further comparative genomics suggests that genetic heterogeneity is present in the three *mcr*-*1*-positive *E. coli* isolates (Fig. 1, S2). We retrieved the sequences of seven house-keeping genes (*adk*, *fumC*, *gyrB*, *icd*, *mdh*, *purA* and *recA*) from the above three sequenced genomes and subjected them to analyses of Multi-Locus Sequence Typing (MLST) (http://mlst.warwick.ac.uk/mlst/dbs/Ecoli). Unlike the epidemic spreading clone, *E. coli* ST131 that carried the *mcr*-*1* gene in Denmark [8], the three *mcr*-*1*-harbouring clinical strains belong to different sequence types (i.e., E15004 is in ST40, E15015 is in ST642, and E15017 is in ST648) (Table 1, Fig. S3), which is generally consistent with our findings from comparative genomics (Fig. 1, S2). The fact that *mcr*-*1*-harbouring *E. coli* isolates are classified into different sequence types argues that the dissemination of *mcr*-*1* colistin resistance gene is ongoing by clonal expansion [9]. Given the fact that *E. coli* ST648 was associated with ESBL [10, 11] and two variants of New Delhi metallo-β-lactamase 1 (NDM-1), NDM-5 [12] and NDM-7 [13]), we thereby were interested in determining whether or not the genes of ESBL and NDM would also be found with the *mcr*-*1* gene in the ST648 strain, E15017.

**Table 1:** Diversified sequence types of the *mcr*-*1*-positive *E. coli* strains revealed by bacterial genomics sequencing

Strains	Alleles							ST	ST Complex
	*adk*	*fumC*	*gyrB*	*icd*	*mdh*	*purA*	*recA*		
MG1655	10	11	4	39	8	8	2	ST98	ST10 Cplx
E15004	6	4	5	26	20	8	14	ST40	ST40 Cplx
E15015	9	23	33	18	11	8	6	ST642	ST278 Cplx
E15017	92	4	87	96	70	58	2	ST648	ST648 Cplx

Genotyping of the *E. coli* strains was conducted through extensive alignments of the seven house-keeping genes (*adk*, *fumC*, *gyrB*, *icd*, *mdh*, *purA* and *recA*) processed with the server of Multi-Locus Sequence Typing (MLST) (http://mlst.warwick.ac.uk/mlst/dbs/Ecoli)

Using ResFinder2.1, a newly-improved database for identifying antibiotic resistance genes (https://cge.cbs.dtu.dk/services/ResFinder), we screened the above three genomic sequences, as well as the remaining unordered contigs, which likely encode additional plasmids, for the presence of antibiotic resistance genes *esp*. ESBL and NDM-1 (and/or its variants). As anticipated, a 100 % identical *mcr*-*1* gene was observed in the unordered contigs in each of the three strains. NDM-1 variants were not found, which we then verified by PCR-based detection (not shown). Unexpectedly, no other antibiotic resistance gene besides *mcr*-*1* is found in the strain E15004 (ST40) (not shown), whereas multiple drug-resistance genes apart from *mcr*-*1* were identified in the unordered contigs from the other two strains, E15015 (ST642) and E15017 (ST648) (Table 2, S2). In particular, the *blaCTX*-*M*-*15* gene that encodes ESBL was found to be present in the ST648 strain, E15017 (Table 2). Additionally, we noted that the *mcr*-*1* and *blaCTX*-*M*-*15* are located inside distinct unordered contigs, suggesting the possibility that they are encoded on different plasmids. This represents the first example of a clinical clone of *E. coli* with a sequence type of ST648 that has the potential to spread MCR-1 colistin resistance together with ESBL resistance.

**Table 2:** Genome-wide screening of the extended-spectrum β-lactamase in the *mcr*-*1*-positive E15017 strain with multidrug resistance genes

Resistance genes	Length (bp)	Contigs	Functions/phenotypes
*aadA5*	789	Contig_13	Aminoglycoside adenyl-transferase AadA5, Aminoglycoside resistance
*strA*	804	Contig_26	Aminoglycoside resistance, aph(3”)-Ib)
*strB*	837	Contig_26	Aminoglycoside resistance, aph(6)-Id
*blaCTX-M-15*	876	Contig_26	Extended-spectrum β-lactamase
*blaTEM-1B*	861	Contig_26	β-lactam resistance
*mph(A)*	906	Contig_13	Macrolide resistance
*sul1*	840	Contig_13	Sulphonamide resistance
*dfrA17*	474	Contig_13	Dihydrofolate reductase DfrA17, Trimethoprim resistance

In summary, our data provides genomic insights into three strains of *mcr*-*1*-positive *E*. *coli* with multiple drug resistance, which reveals the increasing possibility of ST648 becoming an epidemic vector for circulation/spread of the *mcr*-*1* colistin resistance gene in China. As the inter/intra-species dissemination of the *mcr*-*1* gene has been linked to the spread of other drug resistance including ESBL [11] and NDM-1 variants [12, 13], our findings underscore the urgent need to modulate and control the use of colistin in veterinary/clinical practices, which might facilitate prevention of the further emergence of superbugs with multi-drug resistance.

## Electronic supplementary material

Below is the link to the electronic supplementary material.

Supplementary material 1 (PDF 445 kb)
