# Supplementary material for: Genomic insights into the ESBL and MCR-1-producing ST648 Escherichiacoli with multi-drug resistance
Source: Sci Bull (Beijing). 2016 May 19;61:875–8. doi: 10.1007/s11434-016-1086-y (PMC4899488; doi:10.1007/s11434-016-1086-y)
Supplement: Supplementary file 1 — Supplementary material 1 (PDF 445 kb) [file 11434_2016_1086_MOESM1_ESM.pdf]

Supplemental Material

Supplemental tables

Table S1 General features of three *E. coli* genomes

Organization and characterization of the genomes	Strains		
	E15004	E15015	E15017
General features			
Sequenced size (bp)	4,834,707	5,135,345	5,176,582
Assembled size (bp)	**4,643,275**	**4,637,424**	**4,780,540**
GC content (%)	50.49	50.38	50.42
Total number of contigs	59	142	63
Total number of unordered contigs	10	59	18
Function category			
Total number of SNPs	65495	67563	113999
Total number of complete CDS	2593	2607	2502
Total Number of altered CDS	1726	1712	1817
Percent bases missed in reference	9.52%	9.70%	10.93%
Percent bases extra in unordered contigs	10.69%	9.88%	16.74%

Table S2 List of drug resistance genes identified in the *mcr*-1-positive strain E15015

Resistance genes	Length (bp)	Contigs	Functions/phenotypes
*aadA*5	789	Contig_102	Aminoglycoside adenyl-transferase AadA5, Aminoglycoside resistance
strA	804	Contig_84	Aminoglycoside resistance, *aph*(3")-Ib
strB	837	Contig_84	Aminoglycoside resistance, *aph*(6)-Id
blaTEM-1A	861	Contig_84	β-lactam resistance
oqxB	2450	Contig_124	Quinolone resistance
qnrS1	657	Contig_139	Quinolone resistance
floR	1215	Contig_62	Phenicol resistance
sul2	816	Contig_84	Sulphonamide resistance
tet(A)	1172	Contig_84	Tetracycline resistance
dfrA17	474	Contig_102	dihydrofolate reductase DfrA17, Trimethoprim resistance

Supplemental figures

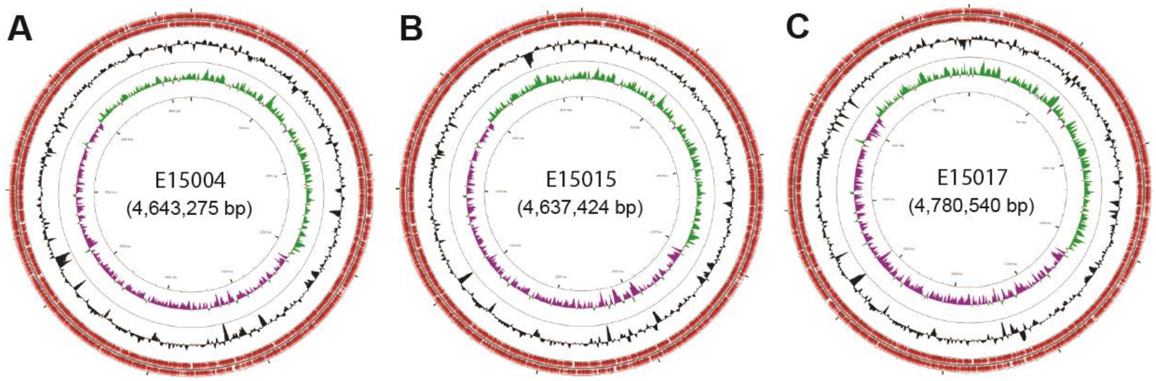

Figure S1 Circular representation of the three assembled genomes (E15004, E15015 and E15017)

The genome maps of E15004 (**A**), E15015 (**B**) and E15017 (**C**) were generated with CGViewer (http://stothard.afns.ualberta.ca/cgview_server/). Individual rings ranging from 1 (inner ring) to 3 (outer ring) separately denote GC Skew (Green: GC skew+; Magenta: GC skew-), GC contents, and putative ORF.

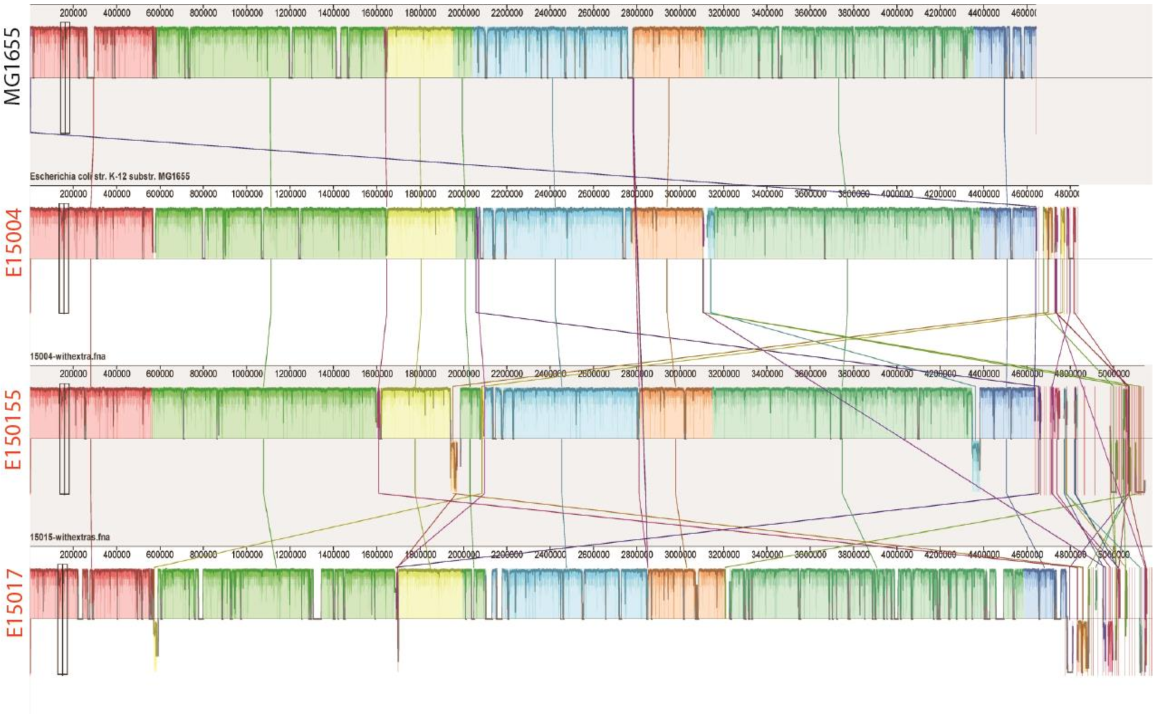

Figure S2 Linear genomic comparison of the three *micr*-1-positive *E. coli* isolates (E15004, E15015 and E15017) to the reference strain MG1655

The colored blocks represent Locally Collinear Blocks (LCBs) which share homology between strains. Lines connecting individual LCBs show their location in the other genomes. Line plot inside each LCB shows local conservation in reference strain. Additional unordered contigs of sequenced genomes appear at right edge.

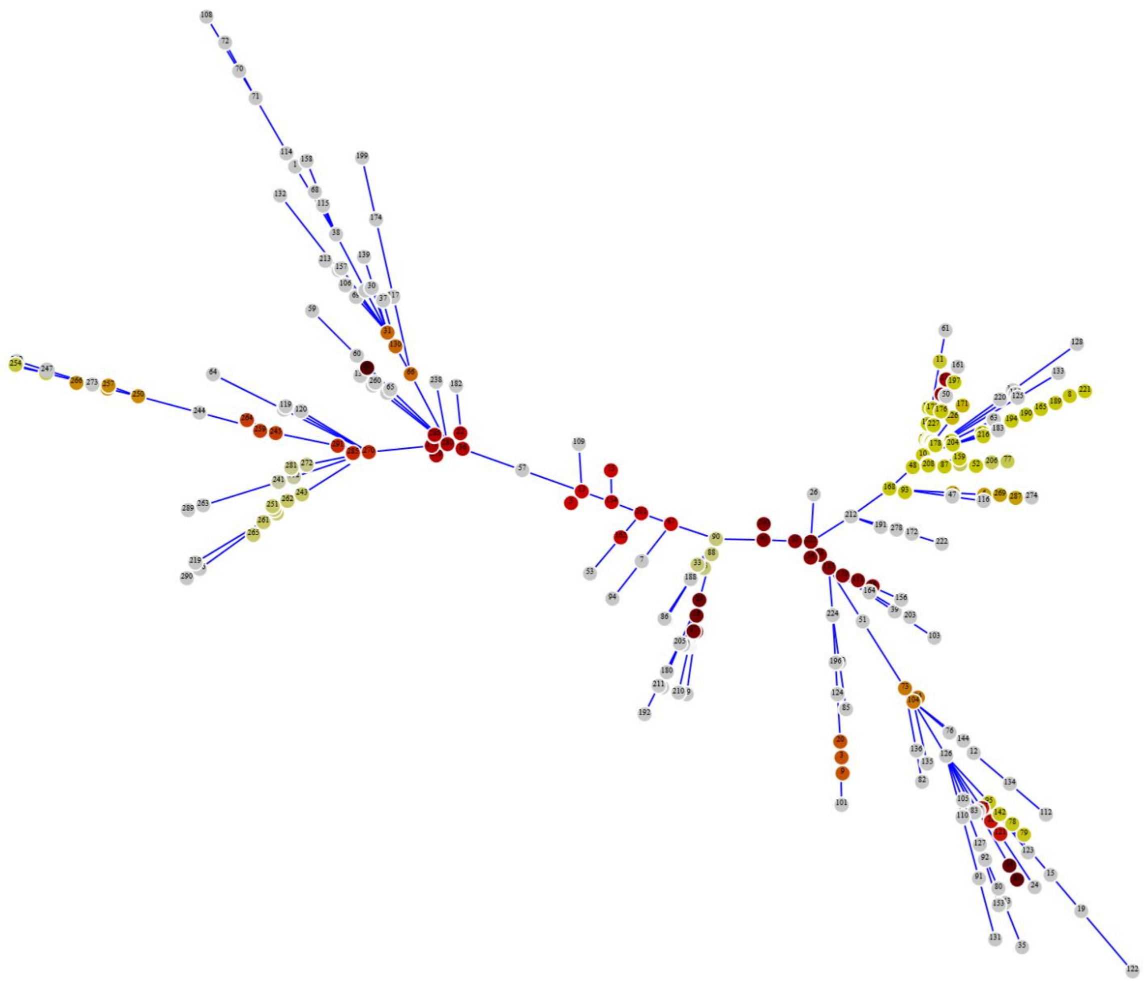

Figure S3 A diagram of sequence typing for the three *mcr*-1-positive *E. coli* isolates (E15004, E15015 and E15017) using the MLST server

The three strains (E15004, E15015 and E15017) were assigned to ST40, ST642, and ST648, respectively.
